# Effects of Technology-Assisted Rehabilitation After Spinal Cord Injury: Pilot Randomized Controlled Crossover Trial

**DOI:** 10.2196/78091

**Published:** 2025-10-02

**Authors:** Mia Maria Kilkki, Joonas Poutanen, Kari Kauranen, Jari Arokoski, Sinikka Hiekkala

**Affiliations:** 1 Department of Public Health, Faculty of Medicine, University of Helsinki, Tukholmankatu 8 B, Helsinki, 00014, Finland, +358 40 5734109; 2 Validia Rehabilitation Ltd, Helsinki, Finland; 3 Faculty of Social Services and Health Care, LAB University of Applied Sciences, Lappeenranta, Finland; 4 Department of Internal Medicine and Rehabilitation, Division of Rehabilitation, Helsinki University Hospital, Helsinki, Finland; 5 Department of Surgery, Faculty of Medicine, Helsinki University Hospital, Helsinki, Finland; 6 Finnish Association of People with Physical Disabilities, Helsinki, Finland; 7 Department of Health Sciences, Faculty of Sport and Health Sciences, University of Jyväskylä, Jyväskylä, Finland

**Keywords:** crossover design, goal attainment scaling, international classification of functioning, disability and health, randomized controlled trial, spinal cord injury, technology-assisted, rehabilitation, robotic, upper extremity

## Abstract

**Background:**

Technology-assisted and robotic rehabilitation methods are increasingly used in neurorehabilitation. Still, scarce evidence exists on their effects on upper extremity functioning after spinal cord injury, especially at the chronic stage.

**Objective:**

The present study aims to evaluate the effects of a 6-week intervention focusing on technology-assisted upper extremity rehabilitation in adults 1-8 years after incomplete cervical spinal cord injury.

**Methods:**

In this pilot randomized controlled crossover trial, 20 participants (10 men, 34‐73 y of age) were recruited by mail and randomized into 2 sequences (AB: n=10 and BA: n=10). All participants received a 6-week rehabilitation intervention in Period 1 or Period 2, with a 4-week washout period in between. The intervention was delivered 3 times a week for 6 weeks (18 sessions) by occupational therapists specialized in spinal cord injuries. Each 1-hour therapy session included a minimum of 30 minutes of technology-assisted upper extremity rehabilitation using interactive and task-specific AMADEO, DIEGO, or PABLO devices. Other occupational therapy activities were allowed to complete the 1-hour therapy session. The effects of the 6-week rehabilitation intervention were compared with 6 weeks of no intervention, and the analyses were based on paired data. Each participant served as their own control. Hand and arm function were evaluated using the Action Research Arm Test, the American Spinal Injury Association–Upper Extremity Motor Score (ASIA-UEMS), grip strength, pinch strength, and the Spinal Cord Independence Measure–Self Report. Rehabilitation goal attainment was evaluated by the Goal Attainment Scale. Face-to-face assessments were conducted at baseline, after Period 1, after Period 2, and at 6 months, except for the Goal Attainment Scale, which was used at the beginning and immediately after the rehabilitation intervention.

**Results:**

The effects of the rehabilitation intervention compared to no intervention were statistically insignificant, except for the ASIA-UEMS (median change 1, IQR 0‐2 point versus 0, IQR −2 to 0 point) in participants in sequence BA (n=7) who received the rehabilitation intervention during Period 2 (*P*=.04). The rehabilitation intervention showed good feasibility and tolerability in adults with incomplete cervical spinal cord injury. Of 20 participants (median age 62, IQR 58‐66 y), 19 enrolled in the study, and 17 completed at least 80% of the rehabilitation sessions. Fourteen out of 16 participants included in the final analysis attained their rehabilitation goals. The goals were mainly focusing on “fine hand use” and “hand and arm use” related to self-care and domestic life.

**Conclusions:**

Results of this pilot study suggest that technology-assisted upper extremity rehabilitation provided by occupational therapists is safe and has potential for broader clinical use in adults with incomplete cervical spinal cord injury. The rehabilitation intervention showed good feasibility and positive outcomes in rehabilitation goal attainment. This study was left unpowered, and the results need to be confirmed in a larger randomized controlled trial.

## Introduction

Spinal cord injuries (SCIs) remain a significant global health concern with high incidence, prevalence, and years lived with disability [1]. In Finland, approximately 500 new cases occur annually [2]. Cervical SCIs are particularly severe, often leading to incomplete or complete tetraplegia. The extent of loss in motor function in the upper extremities depends on the level and completeness of the cervical injury, with higher and more complete injury causing more profound impairments [3].

The loss of upper extremity function is especially debilitating when affecting the ability to perform essential activities related to self-care, domestic life, work, and mobility. Consequently, recovery of upper extremity function has been identified as the highest priority in adults with tetraplegia following SCI [4-6]. Therefore, there is a crucial need for effective rehabilitation methods to restore and improve these essential functions.

The use of technology-assisted and robotic rehabilitation methods is increasing, supplementing conventional neurorehabilitation approaches [7-10]. The primary goal of these methods is to facilitate adaptive plasticity and strengthen intact motor pathways through intensive, repetitive functional training [9,11]. They also facilitate relearning of lost motor skills through the repetition of precise, high-quality movements and activate and strengthen affected muscles.

Robotic rehabilitation shows promise in improving hand and arm function in individuals with stroke [10,12] and SCI [11,13]. Yet, there is scarce evidence, mostly case series, of the effects of technology-assisted upper extremity rehabilitation in individuals with SCI [9,11,14-16]. Only a few pilot randomized controlled trials (RCTs) have evaluated the effects of robotic rehabilitation on hand and arm functions [17-21] and self-reported independence in daily activities [17,18] among individuals with incomplete cervical SCI. Sample sizes have been small, focusing on acute and subacute rehabilitation, and the differences between the groups have been mainly statistically insignificant. We aimed to gain new evidence on the effects of technology-assisted upper extremity rehabilitation in the long term after cervical SCI.

Several studies have recently tested and explored technology-assisted rehabilitation methods in diverse patient populations with chronic health conditions such as Parkinson disease and dementia [22], after stroke [23-25], and in critically ill patients in intensive care units [26], showing good feasibility, usability, and preliminary effects. This study aimed to evaluate the effects of a 6-week intervention focusing on technology-assisted upper extremity rehabilitation in adults with incomplete cervical SCI.

## Methods

### Study Design

This pilot randomized controlled crossover trial (NCT04760470) was conducted following the Consolidated Standards of Reporting Trials extension to crossover trials [27]. The trial duration was 16 weeks, including two 6-week periods (Period 1 and Period 2) and a 4-week washout period in between. The site of intervention, Validia Rehabilitation Center in Helsinki, Finland, is specialized in subacute and later-stage rehabilitation of individuals with SCI.

Participants were randomized into sequence AB or BA in a 1:1 ratio. The sequences were generated prior to the trial using a block design and placed in sealed envelopes in the block size of 6. After the baseline assessment and final eligibility, the sequence-blinded assessor (JP) contacted the rehabilitation site to give the participant’s contact information to the occupational therapist providing the 6-week rehabilitation intervention. The occupational therapist conducted the randomization and then contacted the participant to give further instructions and scheduled 18 therapy sessions. By this way, the randomized sequence was known only to the participants and supervising occupational therapists.

**Figure 1.**
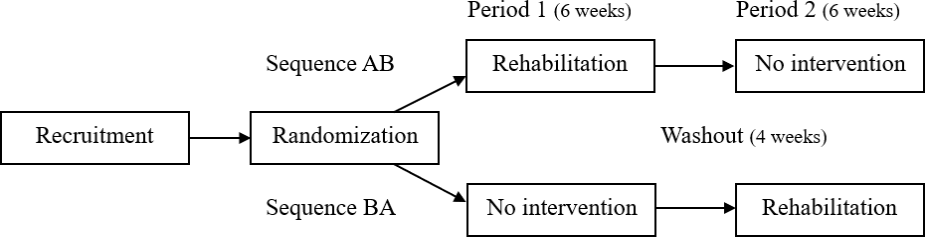
Illustration of this 2-sequence, 2-period randomized controlled crossover trial adapted from Lim et al (2021) [28].

All participants received the intervention. In Period 1, half of the participants (n=10), randomized into sequence AB, received a 6-week rehabilitation intervention, while the other half (n=10), randomized into sequence BA, continued with no intervention. In Period 2, the roles were switched (Figure 1). None of the participants received other physical rehabilitation (physio- or occupational therapy) during the trial.

A crossover design was chosen because of a limited number of trial candidates and their diverse functional abilities, which are typical for individuals with SCI. The design allowed us to compare the treatment effects of a 6-week intervention focusing on technology-assisted upper extremity rehabilitation to no intervention in each participant [28]. Moreover, within-participant comparison reduced the effects of covariates. The target group of the study represented adults with chronic and stable stages of SCI, minimizing the possible period effect. The carryover effect was possible because of the nature of rehabilitation.

### Participants

The target population was recognized from the patient registry of the SCI outpatient clinic of the Helsinki University Hospital by a clinician (JA). Candidates’ neurological level of injury was classified according to the International Standards for Neurological Classification of SCI [29]. Inclusion criteria were 18‐75 years of age, clinically diagnosed with incomplete cervical SCI (American Spinal Injury Association Impairment Scale [AIS] grades C-D), and 1‐10 years since injury.

Individuals with health conditions that could prevent intensive rehabilitation or bias results were excluded by the clinician (JA). Other exclusion criteria were screened during the inquiry calls (SH and JP) and at the baseline assessment by an experienced physiotherapist (JP). Exclusion criteria were injuries preventing training, poor tolerance for intensive rehabilitation, severe instability or severely limited range of motion in upper extremity joints, severe spasticity in upper extremity muscles, cognitive inability to understand the goal and instructions during rehabilitation, concurrent participation in another study or technology-assisted upper extremity rehabilitation, and unlikeliness to complete the intervention and participation until 6-month follow-up.

The patient registry was screened in February 2021, July 2022, and February 2023. In November 2021, due to slow progress in recruitment, the inclusion criterion concerning the time since injury was extended from 1‐5 to 1‐10 years. Altogether, 148 candidates were invited to participate in the study via 6 separate mailings over a 26-month period between February 2021 and April 2023. In addition, information about the study was published in a national SCI patient journal to motivate eligible candidates. One to 2 reminders were sent for candidates without response.

### Ethical Considerations

The technology-assisted upper extremity rehabilitation in subjects with incomplete cervical SCI study was approved by the Ethics Committee of Helsinki University Hospital (669/2020). Written informed consent was obtained from all participants prior to inclusion, and the principles of the Declaration of Helsinki were followed. Participation was voluntary and no compensation was provided. All data were handled confidentially and stored separately from any personal contact information.

### Rehabilitation Intervention

The 6-week intervention focusing on technology-assisted upper extremity rehabilitation was delivered by occupational therapists experienced in treating patients with SCI. The intervention was provided face-to-face, 3 times a week for 6 weeks (18 sessions) in a private outpatient clinic specialized in neurorehabilitation. Each 1-hour therapy session included a minimum of 30 minutes of technology-assisted upper extremity rehabilitation with interactive and task-specific rehabilitation devices AMADEO, DIEGO, and PABLO from Tyromotion GmbH (Graz, Austria) utilizing direct biofeedback and gamification. These 3 devices were chosen as they are specially designed for upper extremity rehabilitation, easy to use, and available at the rehabilitation site involved. The occupational therapists were also familiar with these devices for a longer period of time.

The rehabilitation intervention with progressive intention was personalized on each participant’s individual needs and capabilities. AMADEO allowed training unilateral grasping movements and finger extension passively, actively, and assist-as-needed. DIEGO allowed training unilateral and bilateral arm and shoulder movements in 3D space with intelligent weight relief. PABLO included 3 options for hand and arm rehabilitation. PABLO Multiboard allowed training bilaterally postural control, elbow flexion and extension, as well as upper extremity mobilization in different directions. PABLO Multiball allowed to train wrist movements in all directions, and PABLO Handsensor allowed to train various finger grips and grasp, as well as upper extremity movements in 3 motion axes. Devices were selected individually for each participant based on their functional abilities and rehabilitation goals. Besides the minimum of 30 minutes of technology-assisted rehabilitation, occupational therapists were asked to include other relevant therapy activities based on each participant’s personal rehabilitation goals to complete the 1-hour therapy session. There was no need for content changes during the trial.

### Outcome Measures

Data from all but one outcome (the Goal Attainment Scale [GAS]) was collected face-to-face at baseline, after Period 1, after Period 2, and at 6 months. The effects of rehabilitation intervention on participants in sequence AB were assessed between the baseline and “after Period 1” assessments and on participants in sequence BA between “after Period 1” and “after Period 2” assessments. All outcome assessments (except for the GAS) were conducted by the same sequence-blinded assessor (JP), who was trained for the outcome measures and had no role in implementing the rehabilitation intervention. Measuring points were scheduled in advance based on the baseline assessment. Assessments were conducted at the site of rehabilitation intervention between April 2021 and August 2024. For compliance evaluation, the content of each therapy session was recorded case by case on a study-specific form by the supervising occupational therapist.

Action Research Arm Test (ARAT) was used to evaluate the ability of upper extremity to grasp and transfer objects. It was originally developed for individuals with stroke [30] but also used among individuals with SCI [13,20,21]. The outcome consists of 19 items in 4 subscales including grasp (6 items, 0‐18 points), grip (4 items, 0-12 points), pinch (6 items, 0-18 points), and gross movement (3 items, 0-9 points). Each item is scored from 0 to 3 (0, no movement; 1, movement task is partially performed; 2, movement task is completed but takes abnormally long; and 3, movement is performed normally), total score ranging from 0 to 57. Higher scores mean better performance [31]. The total score can be further divided into 3 disability categories (0‐4, severe; 5‐51, moderate; and 52‐57, mild disability) [32]. The intrarater reliability of ARAT has been found high in individuals with stroke (intraclass correlation coefficient >0.98) [31] and (intraclass correlation coefficient >0.99) [30]. The minimal detectable change in individuals with stroke is suggested to be 5.7 points [33]. Average ARAT total score from right and left upper extremities was used for analysis.

American Spinal Injury Association–Upper Extremity Motor Score (ASIA-UEMS) was used to measure upper extremity muscle strength. It is held as a standardized method to evaluate upper extremity function and classify SCIs [34]. Muscle strength of 5 upper extremity key muscles (elbow flexors and extensors, wrist extensors, finger flexors, and abductors) is tested manually with scaling from 0 to 5 (0, total paresis; 5, normal active movement, complete range of motion against gravity and high resistance in functional position), total score ranging from 0 to 25 per side [35]. Grip strength was measured with the Jamar dynamometer and pinch strength with the Pinch meter. The highest value from 3 attempts of grip and pinch in 3 common positions (2-point, 3-point, and lateral pinch) was recorded. The minimal detectable change for grip strength after stroke is 2.7‐4.7 kg [36].

Functional independence was assessed with the Spinal Cord Independence Measure–Self Report (SCIM-SR) [37]. SCIM-SR is a frequently used patient-reported outcome measure that has shown adequate psychometric properties to assess functioning in daily activities of individuals with SCI [38]. It consists of 17 items in 3 subscales, including self-care (6 items, 0‐20 points), respiration and sphincter management (4 items, 0‐40), and mobility (9 items, 0‐40). Item scores range from 0‐3 to 0‐15, the total score being 0‐100. Higher score equals better performance and independence [39]. The minimal detectable change for SCIM-SR total score is 5.3 and for self-care subscore is 1.5 [40].

GAS is a widely used person-centered and collaborative approach to set personally meaningful goals and evaluate the effectiveness of a rehabilitation intervention [41-43]. The idea is to set a realistic rehabilitation goal using the SMART (Specific, Measurable, Attainable, Realistic, and Timed) principles and then form a personalized 5-point scale, ranging from −2 to +2, to quantify progress toward defined rehabilitation goal [44]. Zero represents the expected level of performance after rehabilitation, +1 represents somewhat more than expected, and +2 represents much more than expected. Respectively, −1 refers to somewhat less than expected and −2 refers to much less than expected (the initial level before the rehabilitation) [42,45-48].

Each participant set their personal rehabilitation goals together with the supervising occupational therapist at the beginning of the rehabilitation intervention. GAS was then used only once after the intervention to evaluate the immediate effects of rehabilitation. The use of GAS and scaling of personal rehabilitation goals made it possible to compare rehabilitation goal attainment among the participants. In cases of multiple goals, the raw scores (−2 to +2) were summed up and then interpreted with a readymade table of *T* scores ranging from 18 to 82. *T* score is dependent on the number of goals. A *T* score of 50 indicates that the expected target level has been attained, a score >50 refers to more than expected, and a score <50 refers to less than expected. Finally, the rehabilitation goals were linked to the International Classification of Functioning, Disability and Health to see which chapters and categories of functioning and disability were meaningful for the participants with incomplete cervical SCI, aiming to improve their hand and arm function [49,50].

Feasibility of the rehabilitation intervention was defined as >60% of participants enrolled with at least 80% of sessions completed and by the compliance of technology-assisted rehabilitation.

### Statistical Analysis

Analyses were performed using the IBM SPSS version 29.0 for Windows (SPPS Inc, Chicago, IL). Because of the small sample size and after visual inspection, nonparametric tests were used. Baseline differences between sequences AB and BA were examined with Fisher Exact and Fisher–Freeman–Halton Exact tests for categorical variables and with Mann-Whitney *U* test for continuous variables. Descriptive statistics were presented in numbers and relative frequencies and as medians and interquartile ranges (IQR) calculated as Tukey’s Hinges.

The effects of rehabilitation intervention on hand and arm function were compared to effects of no intervention based on paired data. Each participant served as their own control following the crossover design [28]. This within-participant difference between rehabilitation and no intervention was tested using repeated measures of the Wilcoxon signed-rank test. Effects of rehabilitation were tested between baseline and “after Period 1” assessments for sequence AB and between “after Period 1” and “after Period 2” assessments for sequence BA. The threshold for significance was set at *P*<.05.

Sufficient sample size for ARAT was determined using the *t* test calculator of the University of California San Francisco, Clinical & Translational Science Institute. As validation studies for ARAT in individuals with SCI were missing, the earlier findings among individuals with chronic stroke were considered. A mean change of 6.1 (SD 5.2) was used as reference (0.8 power, *P*≤.05) [33]. The calculations suggested 13+13 participants. Individuals with SCI commonly have paresis in both upper extremities. Presuming the drop-out rate of 20%, and to account for the more conservative nature of nonparametric testing, the aim was to recruit 30+30 participants.

## Results

### Participants

**Figure 2.**
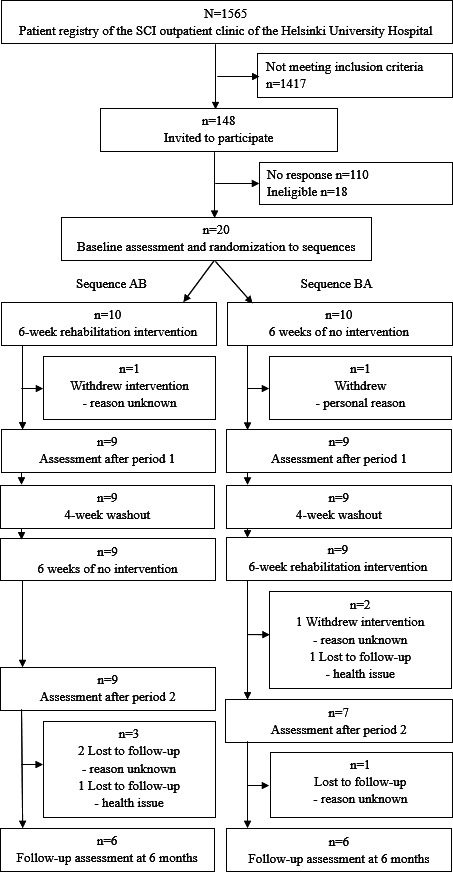
Participant recruitment and retention for the technology-assisted upper extremity rehabilitation in subjects with incomplete cervical spinal cord injury (T-ARSCI) study.

A total of 148 eligible candidates were recognized from the patient registry and invited by mail to participate (Figure 2). Finally, 20 participants, with a median age of 62 years (IQR 58‐66), were recruited and randomized into sequences AB (n=10) and BA (n=10). Nine participants in sequence AB and 8 participants in sequence BA received intended rehabilitation intervention. Participant 10 (in sequence BA) withdrew from the study prior to the trial, believing not to benefit from the rehabilitation intervention. Participants 18 (AB) and 19 (BA) withdrew during the rehabilitation intervention due to personal reasons unrelated to the intervention. Participant 17 (BA) was unavailable for the last 2 assessments because of a health issue. This resulted in a final analysis sample of 16 participants (9 men and 7 women), with a median age of 63 (IQR 54‐66) years. Differences between participants (n=16) and drop-outs (n=4) in age (63, IQR 54‐66 versus 62, IQR 62‐62 years), sex (male 9/16 vs 1/4), and years since injury (3, IQR 2‐7 versus 5, IQR 2‐7) were not statistically significant.

All 20 participants (10 men, 34‐73 y of age; 1‐8 y since SCI) had incomplete cervical SCI graded as AIS D, most of which were caused by a disease (Table 1; Table S1 in Multimedia Appendix 1). The majority of participants had mild upper extremity disability [32] and were able to walk.

Nineteen participants out of 20 (95%) enrolled in the study, and 17 out of 19 (89%) completed at least 80% of the therapy sessions. The number of completed therapy sessions among 16 participants who completed the study was a median of 17 (IQR 17-18) out of 18. Two participants missed 2 sessions, and 8 participants missed 1 session (Table 2). One participant felt pain in the right upper extremity during the rehabilitation intervention. No other side effects nor adverse events were reported.

**Table 1.** Baseline characteristics of 20 participants with incomplete cervical SCI^a^ in total and by sequences in the technology-assisted upper extremity rehabilitation in subjects with incomplete cervical spinal cord injury (T-ARSCI) study.

Variable	Total (N=20)^b^	Sequence AB (n=10)^b^	Sequence BA (n=10)^b^	*P* value
Male, n (%)	10 (50)	10 (50)	10 (50)	>.99^c^
Age (y)	62 (58‐66)	62 (55‐66)	62 (61‐65)	.65^d^
Age at injury onset (y)	58 (53‐61)	56 (53‐62)	59 (55‐60)	.60^d^
Time since injury (y)	3 (2–7)	3 (3–7)	3 (2–7)	.65^d^
Etiology of SCI, n (%)				>.99^c^
Disease	15 (75)	7 (70)	8 (80)	
Trauma	5 (25)	3 (30)	2 (20)	
Level and grade of SCI^e^, n (%)				.13^f^
C2 AIS D	5 (25)	2 (20)	3 (30)	
C3 AIS D	4 (20)	3 (30)	1 (10)	
C4 AIS D	5 (25)	1 (10)	4 (40)	
C5 AIS D	3 (15)	3 (30)	0	
C6 AIS D	2 (10)	—	2 (20)	
C8 AIS D	1 (5)	1 (10)	—	
Mobility outdoors 10‐100 m, n (%***)***				>.99^f^
No devices	12 (60)	6 (60)	6 (60)	
Cane	2 (10)	1 (10)	1 (10)	
Crutches	3 (15)	1 (10)	2 (20)	
Manual wheelchair	1 (5)	1 (10)	—	
Electric wheelchair	2 (10)	1 (10)	1 (10)	
ARAT^h^ total score				
Right	52 (48‐54)	52 (47‐54)	52 (48‐56)	.70^d^
Left	50 (46‐54)	50 (45‐54)	50 (47‐57)	.57^d^
ASIA-UEMS^i^				
Total score	47 (44‐48)	46 (42‐48)	48 (46‐50)	.30^d^
Right	24 (23‐25)	24 (23‐25)	24 (23‐25)	.94^d^
Left	24 (21‐25)	22 (20‐25)	24 (23‐25)	.24^d^
Grip strength (kg)				
Right	29 (24‐36)	33 (25‐35)	25 (16‐36)	.26^d^
Left	26 (16‐32)	25 (15‐34)	26 (16‐30)	.79^d^
Pinch strength (kg)				
2-Point right	4 (4–6)	5 (4–6)	4 (4–6)	.43^d^
2-Point left	4 (2–4)	4 (2–5)	4 (2–4)	.49^d^
3-Point right	6 (5–7)	6 (6–7)	6 (5–6)	.29^d^
3-Point left	5 (4–6)	5 (4–6)	5 (4–5)	.44^d^
Lateral right	7 (6–8)	7 (6–9)	7 (6–8)	.38^d^
Lateral left	6 (4–8)	6 (4–8)	6 (4–8)	.73^d^
SCIM-SR**^j^**				
Total score	90 (78‐98)	88 (71‐98)	97 (80‐99)	.57^d^
Self-care subscore	18 (16‐20)	17 (14‐20)	19 (18‐20)	.26^d^

a SCI: spinal cord injury.

b Reported as median (IQR) unless stated otherwise. IQR=(25th percentile–75th percentile) by Tukey Hinges.

c Fisher exact test (2-sided) *P* value for group difference.

d Mann-Whitney *U* test asymptotic significance (2-tailed) *P* value for group difference.

e According to the International Standards for Neurological Classification of Spinal Cord Injury, expressed by level of injury (C2-C8) and by the American Spinal Injury Association Impairment Scale (AIS, grades A-E, A: complete/no motor, no sensory, no sacral sparing, E: normal/motor and sensory function are normal).

f Fisher–Freeman–Halton exact test (2-sided) *P* value for group difference.

g Not applicable

h ARAT: Action Research Arm Test; total score 0‐57.

i ASIA-UEMS: American Spinal Injury Association–Upper Extremity Motor Score; total score 0‐50 (max. 25 per side).

j SCIM-SR: Spinal Cord Independence Measure—Self Report; total score 0‐100, self-care subscore 0‐20.

**Table 2.** Personal rehabilitation goals and their attainment by GAS^a^ and the change in ARAT^b^ total score before and after the rehabilitation intervention in 16 participants with incomplete cervical spinal cord injury in the T-ARSCI^c^ study.

ID	Rehabilitation goal (ICF code^d^)	GAS *T* score^e^	ARAT right^f^	ARAT left^f^	Sessions^g^
1	It is easier for me to carry and handle a coffee cup (d430, d440)	60	–4	–6	18
2	I can make better use of my left hand when playing the piano (d440, d920)	40	0^h^	0^h^	17
3	I can crochet longer with a thick crochet hook (d445, b740, d920) I can crochet a dishcloth with a thin crochet hook (d440, d920) The pain in my upper limbs has relieved (b280)	59	+1	+1	16
4	It is easier for me to take a bank card out of my pocket with the right hand (d440)	50	+2	0^h^	17
5	It is easier for me to take the cutlery out of the dishwasher (d440, d640)	50	+1^h^	+3	16
6	It is easier for me to button my shirt (d440, d540)	50	0	–3	17
7	Pain no longer interferes with my sleep (b280, b134) I have less pain in my hands (b280)	44	+3	+1	18
8	I can push a stroller with both hands (d445)	50	0^h^	0	17
9	It is easier for me to button my jeans (d440, d540)	60	+3	0	17
11	I can zip up a jacket with both hands (d440, d540)	50	0	+12	17
12	I can lift the dishes up into the cupboard (d430, d640)	50	+2	+2	17
13	I can touch my hair at the back of my head (d445)	60	0	–2	17
14	The pain when doing something disturbs me only half of the time (b280, b160) I can write the diary for up to five minutes at a time (d170, b740)	69	+3^h^	+3^h^	18
15	I can use (squeeze and hold) a steel strapping dispenser with both hands for 11 straps (d445, d430, d740) I don’t need to think about my hands even when they are more spastic (b160)	50	+2	0	18
16	I can button a flannel shirt with six buttons with both hands in less than 2 min (d440, d540) I can knead a liter of bun dough until it is ready for baking. (d445, b740, d630)	62	+2	–3	18
20	I have better activity in my right hand (d445)	60	0	+5	18

a GAS: Goal Attainment Scale.

b ARAT: Action Research Arm Test

c T-ARSCI: technology-assisted upper extremity rehabilitation in subjects with incomplete cervical spinal cord injury.

d International Classification of Functioning, Disability and Health: b134, Sleep functions; b160, Thought functions; b280, Sensation of pain; b740, Muscle endurance functions; d170, Writing; d430, Lifting and carrying objects; d440, Fine hand use; d445, Hand and arm use; d540, Dressing; d630, Preparing meals; d640, Doing housework; d920, Recreation and leisure.

e Goal Attainment Scale *T* score: 50 indicates that the expected level has been attained; >50, more than expected; <50, less than expected.

f Difference before and after the rehabilitation intervention: difference between the baseline and “after period 1” assessments for sequence AB and difference between “after Period 1” and “after Period 2” assessments for sequence BA. Positive score indicates better performance in Action Research Arm Test.

g Total number of therapy sessions during the 6-week rehabilitation intervention (max 18).

h Ceiling effect.

### Implementation of the Rehabilitation Intervention

**Figure 3.**
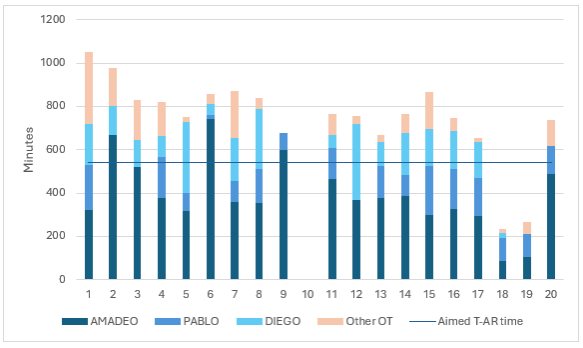
The total time of technology-assisted upper extremity rehabilitation (T-AR) and other occupational therapy (OT) in minutes during the 6-week rehabilitation intervention in 20 participants with incomplete cervical spinal cord injury in the T-ARSCI study. T-ARSCI: technology-assisted upper extremity rehabilitation in subjects with incomplete cervical spinal cord injury.

The aimed minimum of 30 minutes of technology-assisted rehabilitation per session exceeded in all analyzed cases (n=16), the median time being 40 (IQR 38‒44) minutes per session. AMADEO was the most frequently used device (62% of the total technology-assisted rehabilitation time; Figure 3).

Other occupational therapy methods during 1-hour rehabilitation session included stimulation of skin sensation; manual therapy (including fascia manipulation); upper extremity strength training with dumbbells, gym equipment, and other equipment, for example, Reha-Slide Duo (shoulder, elbow, and wrist coordination exercises) and Myro (interactive sensor-based surface); overall relaxation with physioacoustic chair; stretching; spasticity inhibition; paraffin treatment for pain and spasticity management; kinetic taping of fingers; task-oriented functional training; and interview along with goal setting and evaluation.

### Effects of the Rehabilitation Intervention

The effects of the rehabilitation intervention compared to no intervention were not statistically significant, except for the ASIA-UEMS in participants in sequence BA receiving the rehabilitation intervention during Period 2 (Tables S2 and S3 in Multimedia Appendix 1). Median ASIA-UEMS sum score change was 0 (−2 to 0) during no intervention and 1 (0‐2) during rehabilitation intervention (*P*=.04).

Overall, the outcomes varied individually, showing both positive and negative changes before and after the rehabilitation intervention. For example, the average ARAT improved in 4 out of 9 participants in sequence AB and in 6 out of 7 participants in sequence BA (Figure S1 in Multimedia Appendix 1). The greatest improvements in ARAT were observed in the Pinch subscale, as it also showed the most deficits at baseline.

Personal rehabilitation goals were set prior to the rehabilitation intervention, scaled by GAS, and assessed once after the intervention to evaluate the immediate effects of rehabilitation. Goal attainment during the 6-week rehabilitation intervention was mostly successful (Table 2). Fourteen out of 16 participants attained their goals, the median GAS T score being 50 (IQR 50‐60).

**Figure 4.**
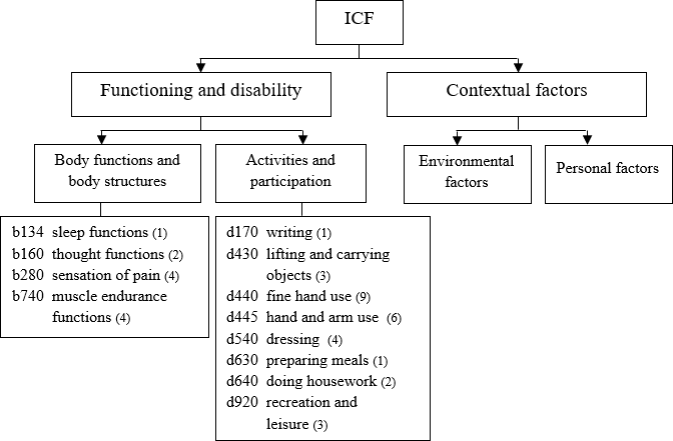
Focus of rehabilitation goals in 16 participants with incomplete cervical spinal cord injury in the T-ARSCI study linked to the International Classification of Functioning, Disability and Health (ICF). Number in parentheses after the ICF category (eg, d170 writing) refers to the number of observed goals. T-ARSCI: technology-assisted upper extremity rehabilitation in subjects with incomplete cervical spinal cord injury.

More than two-thirds of rehabilitation goals were linked to “Activities and Participation” and the rest to “Body Functions and Body Structures” components of the International Classification of Functioning, Disability and Health [45] (Figure 4). “Fine hand use” and “hand and arm use” related to self-care and domestic life were the most targeted categories.

## Discussion

### Principal Findings

The effects of rehabilitation intervention did not differ from the effects of no intervention, except the improvement in upper extremity muscle strength (ASIA-UEMS) in participants randomized into sequence BA. According to GAS, most participants attained their rehabilitation goals focusing on “fine hand use” and “hand and arm use.” Rehabilitation intervention showed good feasibility, compliance, and tolerability in adults with incomplete cervical SCI.

Previous studies have given support for technology-assisted rehabilitation in populations with SCI, highlighting the potential of these technologies to support upper extremity recovery [13,17-19,51,52]. For instance, individuals 2‐8 years after a traumatic cervical SCI at level C4-C6 (AIS grades A-D) benefited from the technology-assisted rehabilitation using wrist robot according to robotic kinematics, although there were no changes in muscle strength of the trained arm [52].

The GAS indicated that a 6-week intervention focusing on technology-assisted upper extremity rehabilitation can address personalized rehabilitation goals effectively. Most goals, such as better handling of a coffee cup, buttoning of the jeans, and ability to prepare meals, were achieved. This aligns with the earlier findings arguing that fine hand use and hand and arm use categories are among the highest priorities among individuals with SCI [53]. Moreover, the intervention was well-tolerated, with only 1 reported side effect (muscle pain) and good compliance among participants. This supports the practicality of incorporating technology-assisted devices into clinical settings, particularly when tailored to individual needs and supervised by specialized occupational therapists.

The variation in within-participant changes during the study may reflect normal fluctuations over time rather than a direct effect of the rehabilitation. In addition, discrepancies between the outcomes and goal attainment by GAS were evident. For future effective studies, it is crucial to reach a bigger sample size, perhaps also a longer period than 6 weeks of intervention. It may also be considered to choose conventional RCT design instead of crossover, despite the diversity of the SCI population and the limited number of study candidates. There are also other spinal cord–specific upper extremity outcomes with proven psychometrics, such as the Graded Redefined Assessment of Strength, Sensation and Prehension [54], to be considered. Although some improvements in hand and arm function were observed over time in both sequences, we could not attribute these changes to the 6-week rehabilitation intervention.

### Strengths and Limitations

This study has both strengths and limitations. Although the crossover design is known to be valuable for small sample sizes and to minimize variability by allowing each participant to serve as their own control, this study was left underpowered. Aimed sample size of 30+30 was not achieved due to challenges in recruitment, including delays caused by the COVID-19 pandemic. The small sample size increases the risk for a type II error, potentially obscuring statistically significant effects. Also, the outcome measures may have lacked the sensitivity to detect subtle changes. For example, 8 participants scored the maximum in ASIA-UEMS already at baseline. Also, the ARAT and the SCIM-SR showed a ceiling effect in this population. Participants were not blinded, which could have biased the outcomes after rehabilitation intervention in favor of higher self-perceived independence by SCIM-SR and better goal attainment by GAS. Finally, improvements observed after rehabilitation in the long term suggest a possible carryover effect.

One strength of the study was randomization that diminished selection bias and ensured balance between the 2 sequences. Another strength was that occupational therapists providing rehabilitation intervention were specialized in neurorehabilitation and SCIs, as well as familiar with the technological devices used. They had no role in recruitment or outcome assessments. Moreover, the sequence-blinded assessor had no role in implementing the rehabilitation intervention. The use of a sequence-blinded assessor ensured the reliability of outcome assessments.

The personalized rehabilitation intervention guided by the SMART goals ensured that the rehabilitation was both meaningful and directly applicable to the participants’ daily life. Furthermore, the intervention demonstrated high feasibility and tolerability, as evidenced by good compliance rates and the absence of major adverse events, supporting its potential for broader clinical implementation.

### Conclusions

Results of this pilot crossover RCT suggest that technology-assisted upper extremity rehabilitation provided by occupational therapists is safe and has potential for broader clinical use in adults with incomplete cervical SCI with mild disability in hand and arm function. Rehabilitation intervention showed good feasibility and positive outcomes, especially in functional goal attainment. However, strong evidence about the effects of the rehabilitation intervention on upper extremity muscle strength was not discovered.

As for future insight, we believe that technology-assisted rehabilitation holds potential to complement neurorehabilitation and may further improve hand and arm function in this population. With a larger RCT, it is possible to refine modern rehabilitation methods to improve hand and arm function and support greater independence in daily life for individuals with SCI.

## Supplementary material

10.2196/78091 Multimedia Appendix 1 Supplementary material, including participant characteristics, within-participant difference in hand and arm function, and Averaged Action Research Arm Test (ARAT) scores.

10.2196/78091 Checklist 1 CONSORT-eHEALTH V1.6.1.

## Abbreviations

AIS: American Spinal Injury Association Impairment Scale
ARAT: Action Research Arm Test
ASIA-UEMS: American Spinal Injury Association–Upper Extremity Motor Score
GAS: Goal Attainment Scale
RCT: randomized controlled trial
SCI: spinal cord injury
SCIM-SR: Spinal Cord Independence Measure–Self Report
SMART: Specific, Measurable, Attainable, Realistic, and Timed
T-ARSCI: technology-assisted upper extremity rehabilitation in subjects with incomplete cervical spinal cord injury
