# Supplementary material for: Effects of Technology-Assisted Rehabilitation After Spinal Cord Injury: Pilot Randomized Controlled Crossover Trial
Source: JMIR Rehabil Assist Technol. 2025 Oct 2;12:e78091. doi: 10.2196/78091 (PMC12490736; doi:10.2196/78091)

**Table S1.** Characteristics of participants (N=20) in the T-ARSCI^a^ study.

| No. | Seq. | Sex | Age^b^ | Level of SCI | ASIA grade | Years since SCI | Mobility device^c^ | ASIA-UEMS^b^ | ARAT^b^ |
| --- | --- | --- | --- | --- | --- | --- | --- | --- | --- |
|  |  |  |  |  |  |  |  |  |  |
| 1 | AB | M | 63 | C2 | D | 3 | 0 | 45 | 54 |
| 2 | BA | F | 47 | C3 | D | 4 | 5 | 50 | 57 |
| 3 | AB | F | 67 | C3 | D | 3 | 2 | 47 | 50 |
| 4 | BA | M | 61 | C6 | D | 2 | 0 | 49 | 46 |
| 5 | BA | F | 65 | C2 | D | 2 | 0 | 50 | 56 |
| 6 | AB | M | 66 | C3 | D | 7 | 3 | 47 | 46 |
| 7 | AB | M | 70 | C2 | D | 6 | 2 | 45 | 44 |
| 8 | AB | F | 34 | C5 | D | 3 | 0 | 41 | 46 |
| 9 | AB | M | 60 | C5 | D | 7 | 2 | 42 | 46 |
| 10^d^ | BA | F | 62 | C4 | D | 7 | 0 | 50 | 57 |
| 11 | BA | M | 64 | C4 | D | 6 | 0 | 47 | 46 |
| 12 | BA | M | 73 | C2 | D | 2 | 1 | 46 | 48 |
| 13 | AB | F | 55 | C4 | D | 2 | 0 | 37 | 54 |
| 14 | BA | F | 53 | C6 | D | 8 | 0 | 46 | 51 |
| 15 | AB | F | 35 | C8 | D | 3 | 0 | 50 | 54 |
| 16 | AB | M | 63 | C5 | D | 1 | 0 | 48 | 48 |
| 17^e^ | BA | F | 62 | C4 | D | 2 | 2 | 37 | 49 |
| 18^f^ | AB | F | 61 | C3 | D | 8 | 4 | 48 | 54 |
| 19^g^ | BA | M | 63 | C2 | D | 3 | 0 | 48 | 50 |
| 20 | BA | M | 66 | C4 | D | 7 | 2 | 42 | 35 |

^a^T-ARSCI: technology-assisted upper extremity rehabilitation in subjects with incomplete cervical spinal cord injury.

^b^At baseline.

^c^Mobility device outdoors within 10–100 meters distance, 0: no device, 1: cane, 2: crutches, 3: manual wheelchair, 4: electric wheelchair, 5: able to move/transfer only assisted.

^d^Participant withdrew after baseline assessment before Period 1.

^e^Participant completed the trial but was not available for the last two assessments for reason unknown.

^f^Participant withdrew from the rehabilitation intervention after 7 sessions for personal reasons.

^g^Participant withdrew from the rehabilitation intervention after 6 sessions for personal reasons.

**Table S2.** Within-participant difference in hand and arm function by Wilcoxon Signed Ranks Test between the rehabilitation intervention and no-intervention in Sequence AB (n=9).

| Outcome | Effects of rehabilitation  (after Period 1 vs. baseline) | Effects of no intervention  (after Period 1 vs. after Period 2) |  |
| --- | --- | --- | --- |
|  | Median (IQR) | Median (IQR) | *P* |
|  |  |  |  |
| ARAT^a^ | 0 (-1–1) | 0 (-1–3) | .11 |
| ASIA-UEMS^b^ | 1 (0–2) | 0 (0–1) | .29 |
| Grip strength (R) | 0 (-2–2) | -2 (-2–1) | .40 |
| Grip strength (L) | 1 (-4–2) | 1 (-1–3) | .34 |
| 2-point pinch (R) | 0 (-1–0) | 0 (0–1) | .23 |
| 2-point pinch (L) | 0 (0–2) | 0 (0–0) | .14 |
| 3-point pinch (R) | 0 (-1–0) | 0 (0–1) | .17 |
| 3-point pinch (L) | 0 (0–2) | 0 (0–1) | .31 |
| Lateral pinch (R) | 0 (0–1) | 0 (0–0) | .34 |
| Lateral pinch (L) | 0 (-1–2) | 0 (0–0) | .49 |
| SCIM-SR^c^ sum | 0 (-4–2) | 1 (0–5) | .40 |
| SCIM-SR^c^ self-care | 0 (0–0) | 0 (0–1) | .71 |

^a^ARAT: Action Research Arm Test, total score 0–57.

^b^ASIA-UEMS: American Spinal Injury Association – Upper Extremity Motor Score, total score 0–50 (max. 25 per side).

^c^SCIM-SR: Spinal Cord Independence Measure - Self Report, total score 0–100, self-care sub score 0–20.

**Table S3.** Within-participant difference in hand and arm function by Wilcoxon Signed Ranks Test between no-intervention and the rehabilitation intervention in Sequence BA (n=7).

| Outcome | Effects of no-intervention  (after Period 1 vs. baseline) | Effects of rehabilitation  (after Period 1 vs. after Period 2) |  |
| --- | --- | --- | --- |
|  | Median (IQR) | Median (IQR) | *P* |
|  |  |  |  |
| ARAT^a^ | 0 (0–3) | 2 (0–3) | .10 |
| ASIA_UEMS^b^ | 0 (-2–0) | 1 (0–2) | .04^c^ |
| Grip strength (R) | 0 (-2–2) | 1 (0–3) | .07 |
| Grip strength (L) | 2 (0–2) | 2 (-2–4) | .92 |
| 2-point pinch (R) | 0 (0–0) | 1 (0–2) | .17 |
| 2-point pinch (L) | 0 (-1–0) | 0 (0–1) | .22 |
| 3-point pinch (R) | 0 (0–1) | 0 (0–0) | .83 |
| 3-point pinch (L) | 1 (0–2) | 0 (0–0) | .20 |
| Lateral pinch (R) | 0 (-2–0) | 0 (0–1) | .07 |
| Lateral pinch (L) | 0 (0–1) | 0 (-1–1) | .39 |
| SCIM-SR^d^ sum | 0 (-6–1) | 0 (-2–2) | .89 |
| SCIM-SR^d^ self-care | 0 (-1–0) | 1 (0–2) | .06 |

^a^ARAT: Action Research Arm Test, total score 0–57.

^b^ASIA-UEMS: American Spinal Injury Association – Upper Extremity Motor Score, total score 0–50 (max. 25 per side).

^c^Statistically significant within participant difference by Wilcoxon Signed Ranks Test.

^d^SCIM-SR: Spinal Cord Independence Measure - Self Report, total score 0–100, self-care sub score 0–20.

**Figure S1.** Averaged Action Research Arm Test (ARAT) score in all four measuring points in 9 participants in Sequence AB and 7 participants in Sequence BA. Sequence AB received rehabilitation during Period 1 and Sequence BA during Period 2. The ARAT maximum total score is 57. Darker bars represent assessments before and after the rehabilitation.


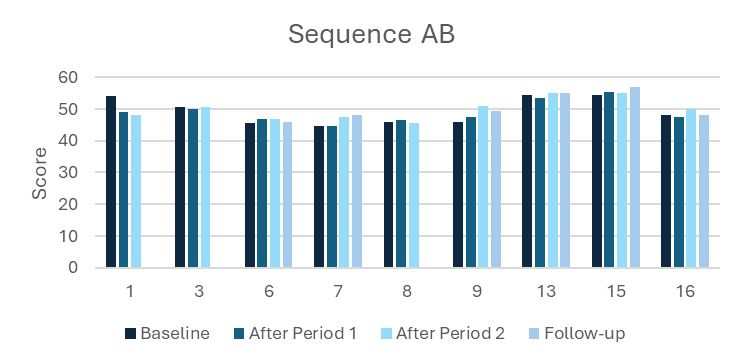


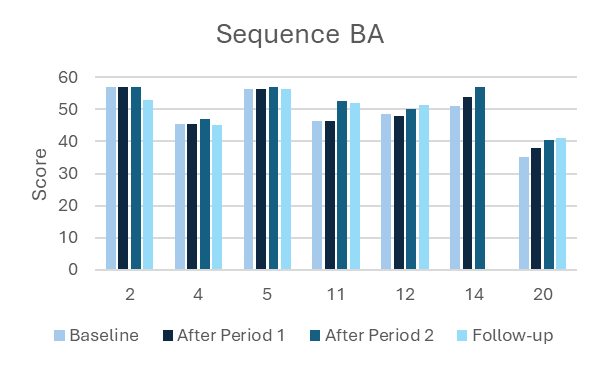

Supplement: Multimedia Appendix 1 [file rehab-v12-e78091-s001.docx]
